# Health literacy and 30-day hospital readmission after acute myocardial infarction

**DOI:** 10.1136/bmjopen-2014-006975

**Published:** 2015-06-11

**Authors:** Stacy Cooper Bailey, Gang Fang, Izabela E Annis, Rachel O'Conor, Michael K Paasche-Orlow, Michael S Wolf

**Affiliations:** 1 Division of Pharmaceutical Outcomes and Policy, UNC Eshelman School of Pharmacy, Chapel Hill, North Carolina, USA; 2 Health Literacy and Learning Program, Division of General Internal Medicine, Feinberg School of Medicine at Northwestern University, Chicago, Illinois, USA; 3 Section of General Internal Medicine, Boston University School of Medicine, Boston, Massachusetts, USA

**Keywords:** CARDIOLOGY

## Abstract

**Objective:**

To assess the validity of a predictive model of health literacy, and to examine the relationship between derived health literacy estimates and 30-day hospital readmissions for acute myocardial infarction (AMI).

**Design:**

Retrospective cohort study.

**Setting and participants:**

A National Institute of Aging (NIA) study cohort of 696 adult, English-speaking primary care patients, aged 55–74 years, was used to assess the validity of derived health literacy estimates. Claims from 7733 Medicare beneficiaries hospitalised for AMI in 2008 in North Carolina and Illinois were used to investigate the association between health literacy estimates and 30-day hospital readmissions.

**Measures:**

The NIA cohort was administered 3 common health literacy assessments (Newest Vital Sign, Test of Functional Health Literacy in Adults, and Rapid Estimate of Adult Literacy in Medicine). Health literacy estimates at the census block group level were derived via a predictive model. 30-day readmissions were measured from Medicare claims data using a validated algorithm.

**Results:**

Fair agreement was found between derived estimates and in-person literacy assessments (Pearson Correlation coefficients: 0.38–0.51; κ scores: 0.38–0.40). Medicare enrollees with above basic literacy according to derived health literacy estimates had an 18% lower risk of a 30-day readmission (RR=0.82, 95% CI 0.73 to 0.92) and 21% lower incidence rate of 30-day readmission (IRR=0.79, 95% CI 0.68 to 0.87) than patients with basic or below basic literacy. After adjusting for demographic and clinical characteristics, the risk of 30-day readmission was 12% lower (p=0.03), and the incidence rate 16% lower (p<0.01) for patients with above basic literacy.

**Conclusions:**

Health literacy, as measured by a predictive model, was found to be a significant, independent predictor of 30-day readmissions. As a modifiable risk factor with evidence-based solutions, health literacy should be considered in readmission reduction efforts.

**Strengths and limitations of this study**

- To the best of our knowledge, this is the first study investigating the association between health literacy and 30-day hospital readmission on a population level, albeit through the use of predictive modelling.
- Results indicate that derived health literacy estimates can be used as basic surrogates of test-based measures to conduct health literacy research on a larger scale than previously possible with direct measures. Conclusions must be tempered, however, due to the use of estimates from a predictive model.
- Agreement between the derived health literacy estimates and individual, test-based measures was fair. Derived health literacy estimates rely on aggregate socioeconomic and demographic characteristics, and are therefore less than ideal.

## Introduction

Nearly 20% of Medicare beneficiaries are readmitted within 30 days of hospital discharge, at a cost approaching US$17.4 billion annually.^1^ The Hospital Readmission Reduction Program (HRRP) was established through the Affordable Care Act, with the objective of reducing readmissions and their associated costs, thereby promoting healthcare quality.^2^ To achieve this objective, the Centers for Medicare & Medicaid Services (CMS) began issuing financial penalties to hospitals with 30-day all-cause readmission rates above the national average for acute myocardial infarction (AMI), heart failure and pneumonia. In FY 2013, more than 2200 hospitals were assessed a total of US$280 million; over the upcoming years the penalty is structured to rise threefold.^3^ Understanding the determinants of readmissions is therefore essential, not only for the financial well-being of healthcare institutions, but also to equitably promote patient safety and healthcare quality.

Health literacy, or one's ability to obtain, process and understand the health information needed to make informed health decisions, is theorised to influence hospital admission and readmissions.^4^ ^5^ According to the 2003 health literacy component of the National Assessment of Adult Literacy (NAAL), over a third of US adults (36%) have *below basic* or *basic* literacy skills, and are likely to have difficulty communicating with providers, interpreting medication instructions, engaging in self-care activities and navigating health systems.^5^ Inability to perform such activities may place patients at an increased risk of rehospitalisation.^6–11^ Yet, unlike other risk factors, health literacy is viewed as a multifaceted and *modifiable* characteristic.^4^ Health literacy-informed interventions have increasingly been shown to significantly improve health outcomes, some even attenuating differences found by literacy.^12–14^ Yet, only a few epidemiological studies to date have identified health literacy as a potential risk factor for hospitalisation.^15^ ^16^ Furthermore, to our knowledge, only two studies have specifically examined the relationship between health literacy and 30-day readmissions; these investigations were conducted among a relatively small sample at local hospitals.^17^ ^18^ As a result, the extent of this association remains unclear. As limited health literacy could be more easily remediated, compared with other patient and system-level factors, and has evidence-based solutions available, understanding this relationship carries significant clinical and policy implications for health systems.

To date, the primary obstacle limiting the study of health literacy for many epidemiological and health services phenomena has been measurement. Current, validated literacy assessments are often time-intensive and logistically challenging, with most requiring in-person administration.^19–21^ An alternative approach has recently emerged, a predictive model of health literacy can be used to estimate the mean health literacy of individuals living in a census tract through US census data.^22–24^ An earlier evaluation of the model found that it was predictive of health literacy as measured by the NAAL, explaining approximately 30% of the variance in average NAAL scores and outperforming single item proxies (ie, education, income).^22^ Additional validation procedures are warranted, however, as the relationship between derived health literacy estimates and more widely used, test-based measures of health literacy is unconfirmed. If validated, the predictive modelling approach could be used universally to explore the relationship between health literacy and a wide range of health outcomes on a larger scale than previously possible with direct assessments. Furthermore, while the predictive model provides only a proxy of health literacy and is reliant on a number of socioeconomic and demographic factors, such variables are often missing, incomplete or inaccurate in large claims data.^25^ As such, analyses of the relationship between these variables and health outcomes are often implausible with these data sets.

In this study, we utilised a unique opportunity to further validate this predictive model as a measure of health literacy. To demonstrate the potential for such a measure, we also sought to investigate the relationship between health literacy and hospital readmissions on a population level. To achieve these aims, we first determined the validity of derived health literacy estimates by examining their association with three commonly used, test-based literacy assessments among a cohort of older patients. We then examined the relationship between the derived health literacy estimates and 30-day hospital readmissions among Medicare enrollees discharged from a hospital stay for AMI in 2008 in Illinois and North Carolina, USA.

## Methods

We conducted a retrospective cohort study utilising data from a National Institute of Aging study entitled ‘Health Literacy and Cognitive Function among Older Adults’ (R01AG030611, referred to as ‘LitCog’),^26^ Medicare Claims and census data. The LitCog study was approved by the Institutional Review Board (IRB) of Northwestern University; the University of North Carolina at Chapel Hill IRB approved Medicare claims and census data analyses.

### Settings and participants

The LitCog cohort was used to assess the construct validity of the derived health literacy estimates by examining their association with three individual health literacy measures. This cohort included English-speaking adults, aged 55–74 years, who were enrolled in the study after receiving medical care at a participating academic general internal medicine clinic or federally qualified health centre in Chicago, Illinois, USA. A full description of the cohort and LitCog study methodology has been previously published.^26^ For current analyses, only those participants who provided a physical, residential address were included in the cohort to enable geocoding.

A Medicare claims-based cohort was then used to assess the association between derived health literacy estimates and 30-day readmission. This cohort included all Medicare beneficiaries who were: (1) ≥65 years old; (2) continuously enrolled in the Medicare fee-for-service and prescription Part D programmes at least 12 months before and until the end of the study period after an index AMI hospitalisation; (3) hospitalised for the index AMI between 1 January 2008 and 31 December 2008, and survived at least 30 days after discharge in Illinois and North Carolina, USA; and (4) were discharged to non-acute care settings. Hospitalisation with AMI was defined as having international classification of diseases (ICD) 9 codes of 410.x1 as the primary or secondary discharge codes in Medicare inpatient claims. The first AMI hospitalisation in the study period was defined as the index hospitalisation for each subject. Data for this cohort were from Medicare service claims and files from the CMS Medicare Chronic Condition Data Warehouse (CCW) from 2007 to 2009.^27^ The CCW files include inpatient, outpatient, skilled nursing facility, physician office visits and prescription Part D event service claims files. All CCW files are linked by an encrypted and unique CCW identifier number for each beneficiary.

### Measures

#### Individual health literacy

Three literacy assessments were administered to patients in the LitCog study during structured, in-person interviews.^26^ The Rapid Estimate of Adult Literacy in Medicine (REALM) is a reading test that assesses patients’ ability to correctly pronounce 66 health-related terms.^21^ The Test of Functional Health Literacy in Adults (TOFHLA) uses a series of short, written instructions and medical forms to measure patients’ reading comprehension and numeracy skills.^19^ The Newest Vital Sign (NVS) tests patients’ ability to navigate and infer written and numerical information provided on a standard nutrition label.^20^ All assessments have been validated, and are three of the most commonly used objective measures in published health literacy research.^28^

#### Health literacy estimates

Health literacy estimates were derived for each census block group in Illinois and North Carolina using the predictive model developed by Martin and colleagues.^22–24^ Martin *et al* used linear regression and data from the 2003 NAAL and 2000 US Census to predict the mean health literacy score of individuals living in a US census tract. The model uses the following predictor variables: gender, age, race/ethnicity, language spoken at home, income, education, marital status, time in the US, and metropolitan statistical area. The mean health literacy scores generated by the model are linked to categories used in the health literacy component of the 2003 NAAL. Specifically, NAAL health literacy scores range from 0 to 500, and are categorised as *below basic* (0–184), *basic* (185–225), *intermediate* (226–309), or *proficient* (310–500). According to the NAAL, individuals with *below basic* or *basic* skills can perform only rudimentary health tasks, such as locating and circling the date on an appointment slip or understanding basic information provided in a health brochure, respectively. Those with *intermediate* skills are able to perform more advanced tasks, such as determining when and how a prescription drug should be taken. Finally, individuals with *proficient* health literacy skills are able to understand more complex and abstract information and perform more complicated health tasks, such as determining health insurance coverage and calculating out-of-pocket costs. More detailed information on both the NAAL and the Martin and colleagues predictive model has been published elsewhere.^5^ ^22–24^

While the original model by Martin *et al*^22^ estimated health literacy at the census tract level, we modified the model to predict health literacy at the census block group level for greater precision. We used the 2010 US Census Summary File 1 to create the gender, age, race/ethnicity variables at census block group level and the 5-year (2006–2010) ACS Summary File to create the variables of language spoken at home, income, education and marital status at census block group level. Time in the USA was entered as a census tract level variable from the ACS Summary File, as no aggregated statistics at block group level were available for this variable. Online supplementary appendix 1 presents the detailed specifications of each variable.

Patients were linked to health literacy estimates by geocoding their residential address and assigning each to a 2010 census block group. The LitCog cohort was geocoded by their complete residential address, and the AMI cohort by their 9-digit, residential ZIP code. For patients who could not be matched to a census block group, we identified their nearest 9-digit ZIP code in our geographic information database by straight-line distance, and assigned the census block group of the nearest 9-digit ZIP code to that subject. Geographic mapping and geocoding was implemented using ArcGIS software (ERSI, Redlands, California, USA). Patient literacy estimates were categorised as *above basic* (score >225) or *basic/below basic* (score ≤225) in accordance with published NAAL categorisation.^5^

#### Hospital readmission

Hospital readmission is defined as any admission, for any cause, to any hospital, within 30 days after discharge from an index AMI hospitalisation. To measure readmission, we used the same criteria utilised by CMS to evaluate hospital performance.^2^ We included measures of whether a patient had a readmission (yes or no) and the total number of readmissions experienced within 30 days postdischarge.

#### Covariates

For the LitCog cohort, patients’ basic sociodemographic information was collected during in-person interviews.^26^ For the AMI cohort, subject's age and gender were collected from the Medicare enrolment file. We measured patient baseline clinical characteristics in the 12 months prior to the index AMI hospital admission and also during the index AMI admission using Medicare CCW medical service files. The 63 clinical characteristics include diagnosis of infection, cancer, diabetes and diabetes complications, dementia and senility, congestive heart failure, and acute coronary syndrome, among others.^29–32^ These variables were measured with the standard algorithms of ICD-9 codes and Condition Categories codes in Medicare medical service files, as used by the CMS for their risk adjustment model.^29–32^

### Analyses

Pearson product-moment and Spearman correlations statistics (literacy measured continuously) and κ agreement tests (literacy measured categorically) were calculated to examine the association between derived health literacy estimates and test-based, individual literacy assessments. Spearman correlation statistics were used for analyses involving the NVS (score range 0–6), while Pearson product-moment correlation statistics were calculated for analyses involving the REALM and TOFHLA (score range 0–66 and 0–100, respectively).

To examine the association between the derived health literacy estimates and 30-day hospital readmission, we first investigated whether the derived health literacy estimate was associated with having a readmission. A multivariable log-binomial regression model was applied to assess the relative risk (RR) of readmission within 30 days (yes vs no) between patients with above basic literacy level and patients with basic/below basic literacy.^33^ ^34^ The models were also adjusted for the clustering of patients within census block groups. Second, we investigated whether derived health literacy estimates were associated with the number of hospital readmissions within 30 days. A multivariable Poisson regression model was applied to assess the incidence rate ratio (IRR) of the number of 30-day hospital readmissions between patients with above basic literacy level and patients with basic/below basic literacy, adjusting for patient clustering within census block groups. Both the log-binomial regression model and the Poisson regression model were analysed by including no baseline characteristics, demographics only, and both demographic and clinical characteristics, respectively. Statistical analyses were performed using SAS V.9.3 (SAS Institute, Cary, North Carolina, USA) and STATA V.12 (StataCorp, College Station, Texas, USA).

## Results

A total of 696 patients were included in the LitCog cohort (table 1). Overall, the mean age of participants was 63 years, 69% were women, and approximately half (54%) were Caucasian. A quarter (24.3%) reported a high school level of education or less, and nearly half (42.5%) had an annual income of less than US$50 000 per year. Literacy skills varied by measure; 21.7% of patients had less than adequate literacy skills according to the REALM, in comparison with 25.7% according to the TOFHLA, and 49.6% according to the NVS.

**Table 1:** Characteristics of the National Institute on Aging Cohort

Variable	All participants (N=6 9 6)
Age (years), mean (SD)	63.2 (5.4)
Female, %	68.5
Race, %	
African-American	37.9
White	54.4
Other	7.7
Education, %	
High school or less	24.3
Some college	20.4
College graduate	21.6
Graduate degree	33.7
Income, %	
<US$10 000	10.4
US$10 000–US$24 999	17.9
US$25 000–US$49 999	14.2
>US$50 000	57.5
Chronic conditions, mean (SD)	1.8 (1.4)

All LitCog patients were matched to a census block group, and a health literacy estimate derived by the predictive model. Using this approach, 31.2% of LitCog patients were estimated to have basic/below basic literacy skills. Table 2 presents the correlations between the individual literacy assessments and the derived estimates among the LitCog cohort. The correlation coefficients were 0.38, 0.42 and 0.50 between the derived health literacy estimates and individual scores on the REALM, TOFHLA, and NVS, respectively (all p<0.001). The agreement between the derived health literacy estimates (above basic vs basic/below basic) and the REALM (≤8th grade reading level vs >8th grade reading level), TOFHLA (limited/marginal literacy vs adequate literacy) and NVS (high likelihood/possibility of limited literacy vs adequate literacy) were 75.5% (κ=0.38, p<0.001), 74.2% (κ=0.37, p<0.001), and 69.8% (κ=0.40, p<0.001), respectively. The levels of agreement between the individual-level measures of health literacy were similar, ranging from 68.7% to 83.4% (p<0.001).

**Table 2:** Association between individual health literacy measures and derived health literacy estimates

Pearson and Spearman correlation tests				
Literacy measures	Derived health literacy estimates	REALM	TOFHLA	NVS
REALM	0.38*†	1.0		
TOFHLA	0.42*†	0.72*†	1.0	
NVS	0.50*‡	0.60*‡	0.64*‡	1.0

κ Agreement test			
Individual literacy measures versus derived health literacy estimates	κ Score	Agreement (%)	p Value
REALM	0.38	75.5	<0.0001
TOFHLA	0.37	74.2	<0.0001
NVS	0.40	69.8	<0.0001

*p<0.001.

†Pearson correlation statistic.

‡Spearman correlation statistic.

NVS, Newest Vital Sign; REALM, Rapid Estimate of Adult Literacy in Medicine; TOFHLA, Test of Functional Health Literacy in Adults.

**Figure 1:**
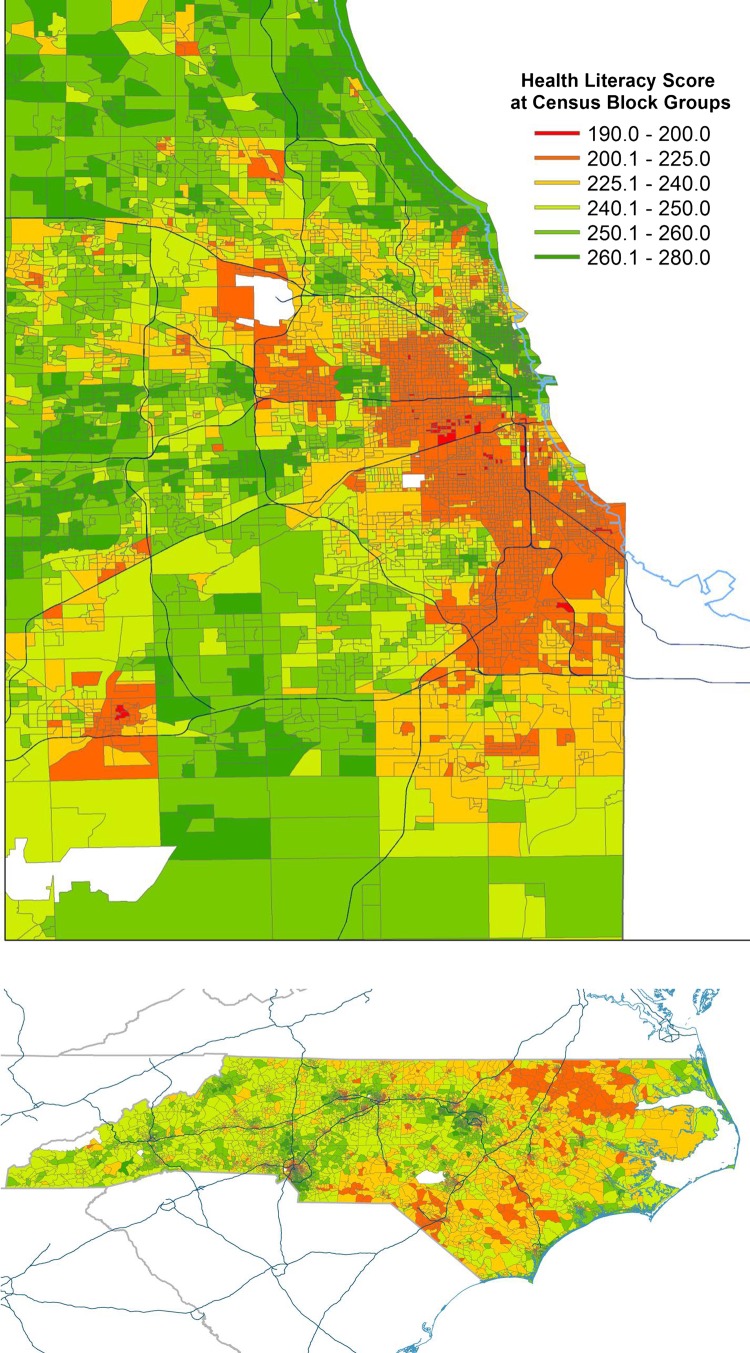
Health literacy estimates across census block groups in Chicago and North Carolina.

Table 3 presents the characteristics of the AMI cohort of Illinois and North Carolina. Approximately 95% of AMI cohort patients were initially matched to a census block. For the remaining 5%, the mean and median distance to the nearest 9-digit ZIP code was 0.06 and 0.02 miles, respectively. Figure 1 shows the variation of derived health literacy estimates across all census block groups in the Chicago metropolitan area and the state of North Carolina. Among the 7733 Medicare AMI patients, 1113 (14.4%) had basic/below basic literacy skills according to the derived health literacy estimates. About 26% of the patients with basic or below basic literacy skills had at least one readmission within 30 days versus 21% of the patients with above basic health literacy skills; there were 33 readmission events per 100 patients with basic/below basic literacy skills in comparison with 25 readmission events per 100 patients with above basic literacy skills, according to the derived health literacy estimates. In general, compared to patients with above basic literacy, patients with basic/below basic literacy had more medical conditions and chronic comorbidities, placing them at greater risk for readmission. For example, 55% of patients with basic/below basic health literacy according to the derived health literacy estimates had diabetes and diabetes complication diagnoses versus 44% of patients with above basic literacy, and approximately 45% of patients with basic/below basic health literacy had congestive heart failure versus 36% of patients with above basic literacy.

**Table 3:** Characteristics of patients discharged for acute myocardial infarction hospitalisation in 2008 in Illinois and North Carolina

Characteristics	Basic/below basic health literacy	Above basic health literacy	Absolute standardised difference*
N (%)	1113 (14.4)	6620 (85.6)	–
*Having hospital readmission within 30 days postdischarge, %*	25.9	21.1	11.4
Number of hospital readmissions within 30 days postdischarge			
Number/100 patients	33.2	25.3	†
Categories, %			
0	74.1	78.9	11.3
1	19.9	17.5	6.2
2+	6.0	3.6	11.2
Demographics, %			
Age group 65–74	42.0	33.5	17.6
Age group 75–84	34.2	38.9	9.8
Age group 85+	23.8	27.6	8.7
Gender: male	36.4	41.7	10.9
Race: white‡	43.8	90.1	112.9
Race: black‡	51.3	7.2	110.9
Race: Hispanic‡	2.6	0.7	14.7
Race: Asian‡	1.1	1.0	1.1
Race: Other‡	1.1	1.0	1.5
Clinical characteristics as 12-month baseline, %			
AMI: anterior	1.9	2.1	1.5
AMI: other location	1.5	2.1	4.1
CABG	4.9	1.7	17.5
PCI	3.6	3.3	1.6
Infection	33.2	27.9	11.5
Metastatic cancer and acute leukaemia	1.3	2.1	5.9
Cancer	20.3	20.1	0.5
Diabetes and diabetes complications	54.5	44.7	19.8
Protein-calorie malnutrition	4.0	2.3	10.3
Disorders of fluid/electrolyte/acid-base	35.8	28.7	15.2
Iron deficiency and other anaemias and blood disease	47.4	36.4	22.5
Dementia and senility	21.9	17.4	11.3
Hemiplegia, paraplegia, paralysis, functional disability	11.6	6.3	18.4
Congestive heart failure	45.2	36.0	18.7
Acute coronary syndrome	25.7	22.7	6.9
Angina pectoris/old myocardial infarction	19.9	20.0	0.2
Coronary atherosclerosis/other chronic ischaemic heart disease	55.9	51.0	9.8
Valvular and rheumatic heart disease	21.5	20.8	1.5
Arrhythmias	37.6	35.8	3.8
Stroke	16.1	9.9	18.6
Cerebrovascular disease	26.1	22.6	8.1
Vascular or circulatory disease	48.2	41.0	14.4
COPD	26.2	27.6	3.0
Asthma	10.5	7.1	12.2
Pneumonia	21.7	19.9	4.4
End-stage renal disease	7.4	2.1	25.0
Renal failure	31.4	22.2	20.9
Other urinary tract disorders	25.0	23.6	3.3
Decubitus ulcer or chronic skin ulcer	11.5	8.4	10.5
Clinical characteristics during Index AMI admission,%			
AMI: anterior	6.4	7.9	5.8
AMI: other location	8.4	12.1	11.9
PCI	27.1	34.5	16.0
CABG	6.3	6.0	1.0
Infection	5.6	4.5	4.9
Metastatic cancer and acute leukaemia	0.6	1.0	4.4
Cancer	3.7	4.3	3.3
Diabetes and diabetes complications	35.2	29.4	12.4
Protein-calorie malnutrition	2.4	2.2	1.7
Disorders of fluid/electrolyte/acid–base	23.2	19.0	10.3
Iron deficiency and other anaemias and blood disease	21.2	17.9	8.2
Dementia and senility	8.0	7.8	0.8
Hemiplegia, paraplegia, paralysis, functional disability	6.4	2.9	16.8
Congestive heart failure	44.2	38.0	12.6
Acute coronary syndrome	0.6	0.9	2.7
Angina pectoris/old myocardial infarction	6.6	6.8	1.1
Coronary atherosclerosis/other chronic ischaemic heart disease	61.2	66.9	12.0
Valvular and rheumatic heart disease	8.4	11.3	9.9
Arrhythmias	32.3	35.3	6.3
Stroke	2.1	1.9	1.1
Cerebrovascular disease	4.9	3.9	4.8
Vascular or circulatory disease	17.9	17.3	1.5
COPD	16.5	17.8	3.3
Asthma	2.5	1.8	5.2
Pneumonia	14.0	13.6	1.3
End-stage renal disease	2.5	0.6	15.2
Renal failure	32.6	23.8	19.6
Other urinary tract disorders	4.5	4.8	1.6
Decubitus ulcer or chronic skin ulcer	3.8	2.2	9.4

*An absolute standardised difference >10 (approximately equivalent to p<0.05) indicates significant imbalance of a characteristic.

†p<0.001, two-sample t test.

‡Race variables reported for informational purposes only. Consistent with CMS hospital readmission risk adjustment modelling methodology, they are not included as covariates in the models.

AMI, acute myocardial infarction; CABG, coronary artery bypass surgery; COPD, chronic obstructive pulmonary disease; PCI, percutaneous coronary intervention.

The results from the regression models are shown in table 4. In the model without adjustment, patients with above basic health literacy according to the derived health literacy estimates had an 18% lower risk of having a 30-day readmission (RR=0.82, 95% CI 0.73 to 0.92) and 23% lower incidence rate of 30-day readmissions (IRR=0.79, 95% CI 0.68 to 0.87) as compared to patients with basic or below basic literacy skills. Adjusting for age and gender did not attenuate this association. When both patient demographic and all 63 clinical characteristics were included in multivariable models, the difference in risk for 30-day readmission was 12% lower (adjusted risk ratio 0.88; 95% CI 0.79 to 0.99) and the incidence rate difference for 30-day readmissions was 16% lower (adjusted IRR 0.84; 95% CI 0.74 to 0.95) for patients with above basic health literacy.

**Table 4:** Relative risk and incidence ratio of 30-day hospital readmission between patients with above basic versus basic/below basic levels of health literacy

Health literacy estimates (above basic vs basic/below basic)	Model 1: no covariates	Model 2: adjusting for age and gender only	Model 3: adjusting for age, gender and clinical risk factors
*Relative risk* of having a 30-day readmission (95% CI), p value	0.816 (0.729 to 0.914), <0.0001	0.814 (0.727 to 0.911), <0.0001	0.882 (0.788 to 0.987), 0.029
*Incidence rate ratio* of 30-day readmissions (95% CI), p value	0.768 (0.676 to 0.872), <0.0001	0.766 (0.674 to 0.871), <0.0001	0.837 (0.737 to 0.951), 0.006

## Discussion

Our results indicate that derived health literacy estimates can be used as proxies for test-based measures to conduct health literacy research on a larger scale than previously feasible with direct assessments. Using derived health literacy estimates at the census block group level, our findings suggest that health literacy is a significant, independent predictor of having a readmission within 30-days of discharge from a hospital stay for AMI; it is also predictive of the number of readmissions experienced by a patient within this timeframe. To our knowledge, this is the first study investigating the association between health literacy, albeit a derived estimate, and 30-day hospital readmission on a population level.

Agreement between the derived health literacy estimates and individual, test-based measures was fair, but less than ideal. This is understandable, as the estimates are based on a neighbourhood average and not individual performance. Interestingly, the levels of agreement between the three individual-level measures of health literacy were comparable with those between the individual measures and the predictive model. This suggests that even among accepted, widely used health literacy measures, variation and measurement challenges exist. While these methods will not be suitable for all health literacy studies, a neighbourhood-based estimate is appropriate for the hypotheses we tested and is the only feasible approach at this time. Other alternatives, such as directly assessing the relationship between hospital readmissions and demographic and socioeconomic factors using Medicare data, is implausible, as claims data often lacks key variables (eg, income, language spoken); furthermore, sociodemographic variables that are available can be inaccurate (ie, race/ethnicity).^25^ This study provides further support for the predictive validity of a derived health literacy estimate, while adding greater precision to prior approaches by using data from census block groups as opposed to census tracts.^22^

It is necessary to note, however, that using a derived health literacy estimate is not without its limitations. Socioeconomic and demographic characteristics that were included in the derived estimate have been shown in prior studies to be independently associated with readmissions, and are also well known to be strongly associated with existing, objective measures of health literacy. Yet, the relationship between socioeconomic and demographic factors and health literacy is so strong—and is the reason for their summative inclusion in the derived variable—that it has not been possible to untangle these factors from health literacy itself. As the Institute of Medicine, WHO, and many other professional societies to date have reiterated, low health literacy is, in fact, often the result of low education, poor opportunity, and limited healthcare access.^4^ ^35^ Our results must also be tempered by the reality that our health literacy estimates are based solely on a compilation of sociodemographic variables, and cannot reflect the full range of individual capabilities encompassed within the concept of health literacy. However, this is often a criticism of other frequently used, direct literacy assessments, which rarely, if ever, assess all the requisite skills needed to obtain, process and understand health information.^24^

Despite these limitations, our study offers some novel insights worth further examination. Our results are particularly timely given recent attention placed on hospital readmissions as a patient safety and healthcare quality concern.^2^ From a payer perspective, the potential that health literacy—a modifiable risk factor—is associated with a 16% lower rate of hospital readmission is remarkable. Future studies are needed to confirm these findings and explore the exact mechanisms through which health literacy may impact this important health outcome. By more fully understanding the role played by health literacy, resources could be better utilised, and evidence-based practices identified to improve patient education and care. For example, if our results are replicated in other studies, derived health literacy estimates could be utilised in national policies and programmes, such as the Health Plan Employer Data and Information Set (HEDIS) measures and the implementation of the ‘Get With The Guidelines’ programme, to tailor education and promote quality of care at hospital discharge.^36–38^ From a public health viewpoint, mapping health literacy estimates could also provide a new means by which to identify neighbourhoods at risk and to better target appropriate interventions for these communities and the hospitals that serve them. This is essential, as currently there is little guidance provided to underperforming hospitals on how to reduce readmissions.

Finally, our findings have implications for CMS policies. Currently, adjusted rates used by CMS to determine financial penalties include only medical comorbidity and age.^2^ However, our findings indicate that health literacy, albeit measured by a predictive model, is also a significant risk factor for readmission. Our study also demonstrates that such analyses can be operationalised on a large scale. The lack of adjustment by CMS for health literacy may place an undue burden on hospitals that serve patients who are at greatest risk for low literacy and are more likely to struggle with self-care activities postdischarge. By not considering health literacy, the current CMS approach may result in financial penalties for hospitals that have the greatest need for resources to serve vulnerable patients, further exacerbating disparities.^39^ ^40^ In response, it may be appropriate for CMS to alter their readmission penalty scheme to include a derived health literacy measure and then to mandate implementation of health literacy-informed interventions in hospitals that are shown to have a greater number of readmissions attributable to limited health literacy. Effectively, averted financial penalties could be earmarked for health literacy interventions to improve care transitions.

Our study has several limitations beyond those associated with our derived health literacy estimates. First, the NIA study cohort used to assess the validity of the health literacy predictive model consisted of older, English-speaking adults in the Chicago metropolitan area who were predominately Caucasian or African-American. Therefore, our results may not be generalisable to younger populations, to individuals who do not speak English, to other racial/ethnic minority groups, or to those living in non-urban areas. Similarly, our analyses of the relationship between derived health literacy estimates and hospital admission were conducted among elderly Medicare beneficiaries, so results may not be generalisable to younger populations. While future research can extend our methods to younger populations, the elderly carry a disproportionate burden for hospitalisation and readmission and is the appropriate focus of this research. Second, the effects of health literacy on readmissions may vary for conditions other than AMI. Future research is necessary to examine effects of health literacy on other conditions such as heart failure and pneumonia. Third, we used Medicare medical service claims to assemble our study cohort and measure baseline clinical risk factors. It remains possible that not all diagnoses were captured in these data. To address those limitations, we applied validated algorithms to identify AMI and medical adjustment variables.^30–33^ ^41–43^

These methods open the door to mapping health literacy on a population level. Further, by applying this approach to the outcome of 30-day readmissions, we provide an example of how such data can be used to inform an active target for policy and health services research. Our results indicate that health literacy as measured by a predictive model is an independent predictor for both the risk and incidence rate of readmission after AMI. Future research is warranted to evaluate the generalisability of these findings to younger patients, other diagnoses, and other states in the USA, as well as to evaluate the efficacy of interventions to ameliorate the impact of health literacy.
